# The Evolution of the Spatial Association Effect of Carbon Emissions in Transportation: A Social Network Perspective

**DOI:** 10.3390/ijerph16122154

**Published:** 2019-06-18

**Authors:** Fei Ma, Yixuan Wang, Kum Fai Yuen, Wenlin Wang, Xiaodan Li, Yuan Liang

**Affiliations:** 1 School of Economics and Management, Chang’an University, Xi’an 710064, China; mafeixa@chd.edu.cn (F.M.); 2016123084@chd.edu.cn (W.W.); 2016123083@chd.edu.cn (X.L.); 2017123045@chd.edu.cn (Y.L.); 2 Department of International Logistics, Chung-Ang University, Seoul 06974, Korea; yuenkf@cau.ac.kr

**Keywords:** transportation carbon emission, gravity model, social network, QAP regression analysis

## Abstract

The association effect between provincial transportation carbon emissions has become an important issue in regional carbon emission management. This study explored the relationship and development trends associated with regional transportation carbon emissions. A social network method was used to analyze the structural characteristics of the spatial association of transportation carbon emissions. Indicators for each of the structural characteristics were selected from three dimensions: The integral network, node network, and spatial clustering. Then, this study established an association network for transportation carbon emissions (*ANTCE*) using a gravity model with China’s provincial data during the period of 2007 to 2016. Further, a block model (a method of partitioning provinces based on the information of transportation carbon emission) was used to group the *ANTCE* network of inter-provincial transportation carbon emissions to examine the overall association structure. There were three key findings. First, the tightness of China’s *ANTCE* network is growing, and its complexity and robustness are gradually increasing. Second, China’s *ANTCE* network shows a structural characteristic of “dense east and thin west.” That is, the transportation carbon emissions of eastern provinces in China are highly correlated, while those of central and western provinces are less correlated. Third, the eastern provinces belong to the two-way spillover or net benefit block, the central regions belong to the broker block, and the western provinces belong to the net spillover block. This indicates that the transportation carbon emissions in the western regions are flowing to the eastern and central regions. Finally, a regression analysis using a quadratic assignment procedure (QAP) was used to explore the spatial association between provinces. We found that per capita gross domestic product (GDP) and fixed transportation investments significantly influence the association and spillover effects of the *ANTCE* network. The research findings provide a theoretical foundation for the development of policies that may better coordinate carbon emission mitigation in regional transportation.

## 1. Introduction

Global attention is currently focused on carbon emissions and climate change. According to the International Energy Agency (IEA), carbon dioxide levels rose by 1.4% in 2017, reaching a record high of 32.5 billion tons. Global oil demand has increased by 1.5 million barrels per day, 60% of which comes from Asia. In recent years, China’s rapid development has stimulated energy demands in the transportation industry [1]. The transportation industry has become the main sector driving China’s energy consumption and is focusing on energy conservation and emissions reduction. In the past two years, the State Council of China has implemented some policies for developing low-carbon transportation systems.

This background highlights the importance of further studying the characteristics of China’s provincial transportation carbon emissions. Carbon mitigation measures have been implemented in different provinces, and the status of the provinces in the carbon emission system and their associated relationship determine the amount of provincial carbon emission reduction [2]. However, the spatial association of transportation emissions between provinces is very complicated. If we only consider the energy consumption and transportation carbon emissions of a single province in isolation, we might miss the intrinsic interactions between the provinces. In fact, the interaction of economic development, energy structure, location, and transportation infrastructure between the provinces can strengthen the spatial heterogeneity of China’s transportation carbon emissions. This highlights the complexity in spatial association structure and the potential synergies of reducing regional transportation carbon emissions [3]. Therefore, it is important to fully understand the spatial network structure of provincial carbon emissions, and to consider both the amount of emissions mitigation and the relationships between the provinces [4]. There are significant differences in energy consumption between the provinces. The differences in transportation carbon intensity cause the inter-province relationships to be complex with respect to transportation carbon flow. Hence, determining ways to explore the association effects and the factors influencing those effects is the first step in realizing carbon emission reduction targets in complex transportation flows. Analyzing spatial association characteristics and the evolution of those characteristics is key to assessing the results of regional ecological civilization. It also provides a scientific basis for achieving low-carbon development.

The most common method for studying complex relationships is social network analysis (SNA). SNA expresses complex associations using an association matrix. Structural features are measured using graph theory and algebraic operations to reflect the key features in complex networks [5]. In the social network analysis, the network is analyzed through three levels: Whole, individual, and sub-group, which not only reveals the network association characteristics from the perspective of attribute data, but also discovers the relationship between network nodes from the perspective of relational data. In the social network analysis, the quadratic assignment procedure (QAP) algorithm studies the correlation between two variable matrices or the regression relationship between multiple variable matrices and a variable matrix by matrix row and column permutation. As network data often suffer from issues, such as multicollinearity and autocorrelation, the QAP algorithm is more suitable for the regression analysis of influencing factors than other methods. In this study, we studied the evolutionary laws associated with the spatial association effect of transportation carbon emissions in China using SNA theory. First, three characteristics were selected to evaluate the transportation carbon emission network: The integral network, node network, and spatial clustering. Second, we obtained transportation carbon emission data from all provinces using a top-down calculation method. We then determined the carbon emission association network and analyzed the evolutionary trend of the network using a gravity model. Finally, we analyzed the spatial association effect of the whole network based on changes in the association diagram. Provincial transportation carbon emissions may be affected by the neighboring provinces. As such, QAP regression analysis was applied to further explore the factors influencing transportation carbon emissions.

The rest of this paper is organized as follows. Section 2 is the literature review of related research. Section 3 constructs the research model and describes the variable selection. Section 4 analyzes the evolutionary trends of the carbon emission network using the research results obtained from analyzing the spatial association effect in China’s provincial transportation. This section also explores the influencing factors of transportation carbon emissions by QAP. The last section summarizes the full study and proposes corresponding policy recommendations.

## 2. Literature Review

Existing carbon emissions research can be grouped into three categories: (1) Calculation and influencing factor analysis; (2) efficiency analysis; and (3) spatial-temporal evolution and prediction. The methods used to calculate transportation carbon emissions mainly include “top-down” and “bottom-up” approaches. For example, Wu et al. [6] used the “top-down” method to measure the carbon dioxide emissions of Gansu Province from 2000 to 2013. In another study, Chen et al. [7] calculated and obtained the carbon emissions of urban traffic in Shanghai from 2000 to 2007, using the number of vehicles, mileage, energy consumption structure, and carbon emission factors for different energy sources.

In addition, time series measurement and spatial measurement model methods are mainly used to analyze factors contributing to transportation carbon emissions. For example, Liao et al. [8] used the time series measurement method to study the influencing factors of carbon emissions of inland waterway transportation in Taiwan. Liu et al. [9] used a spatial auto-association analysis and a spatial econometric model to analyze the spatial dependence of carbon emissions from transportation between provinces in China from 1997 to 2012. Gonzalez [10] studied the energy consumption and carbon emissions of road transport in different countries. Stokenberga [11] and Hillsman [12] conducted carbon footprint calculations for road transport in the country of Mexico and the state of Florida (United States) in their studies. Both concluded that carbon emissions showed an upward trend as energy consumption and passenger traffic increased over time.

Most research has mainly used the data envelopment analysis (DEA) method to calculate carbon emissions efficiency and predictions. For example, Los and Verspagen [13], Hyun-Jin Kim et al. [14], and Hampf and Krüger [15] applied the DEA method to analyze the carbon emission efficiency of the automotive industry in the United Kingdom, Spain, and Germany, respectively. Wang and He [16] used the global DEA method to study the productivity, economic efficiency, carbon emission efficiency, and marginal abatement costs of China’s regional transportation sector from 2007 to 2012. The Laspeyres model and the exploratory spatial data analysis (ESDA) method are mainly used to explore carbon emission intensity and temporal-spatial evolutions. Ryan [17] used panel data to identify a negative correlation between the European Union (EU) vehicle energy tax and carbon emission intensity. Obas et al. [18] used the Laspeyres model to analyze the association between carbon intensity and economic structure, energy type, and energy intensity. Yang and Ning [19] combined the ESDA method with the geographic information system (GIS) spatial analysis method to explore the spatial differences and spatial aggregation laws associated with carbon emissions in China’s transportation system. In summary, research about transportation carbon emissions has mainly focused on the above dimensions described in the studies above. Few studies have focused on the associated relationship of transportation-driven carbon emissions between provinces.

Social network analysis (*SNA*) is a method based on graph theory, which uses relational data to study spatial network associations and the structure of a system. In recent years, many scholars used the SNA method to analyze the spatial network relationship between cities or industries in the field of carbon emissions. For example, Zhao and Yan [20] studied the network characteristics associated with the structure of industry carbon flows by constructing a mobile network of China’s carbon emission industry using SNA methods. They discovered that China’s inter-industry carbon mobility network is a network with small world effects and scale-free characteristics. Scale-free and small world effects are the two most typical characteristics of social networks. The small world effect reflects the closeness of the connections between the various nodes of the network, and scale-free is an intrinsic property that describes the severe uneven distribution of the integral network. Zhou et al. [21] studied 37 cities in the Yangtze river economic belt in 2014, constructing a spatial association network of urban carbon emissions by integrating geographical distance, economic level, population size, carbon emissions, and other factors. They concluded that the density of the urban carbon emission spatial correlation network in the Yangtze River Economic Belt is not very high, and the energy circulation efficiency is low. Prell and Fen [22] used SNA and a multiregional input–output analysis to track how consumption in the United States triggered value-added and carbon inequalities around the globe. They found that commodity production for US consumption tends to reify inequalities between countries.

The studies described above mainly focus on analyzing energy savings and reduced emissions in local areas using microscopic perspectives. Further, studies have generally used attribute data (i.e., characteristics of the spatial entity) rather than “relationships data” (i.e., contacts, group dependencies, and gatherings of spatial entities). Few studies have focused on the spatial-temporal evolution and differences of transportation carbon emissions from an overall spatial perspective. This limits the existing available research on spatial association effect analysis. The theory of spatial interaction holds that there is always an exchange of material, energy, people, and information between cities for the normal operation of production and life. The transportation network itself interacts inter-regionally. The spatial interaction of transportation pollutant emissions and its influencing factors can be collected by analyzing the migration of inter-regional labor, the transportation of goods, the flow of funds, and information delivery. Traditional econometric models do not consider spatial factors, leading to errors in the measurement of influencing factors. Past analyses of the spatial associations of transportation carbon emissions using spatial measurements have been limited to “adjacent” or “close” areas. However, geographically non-adjacent areas may also have correlated carbon emissions. Therefore, a coordinated governance mechanism across China’s provinces, creating an overall network structure of national transportation carbon emissions, is needed.

This study explored the network characteristics associated with the spatial association effect in China’s provincial transportation carbon emissions based on the social network analysis method. The results will help decision makers understand the overall structure of carbon emissions and better understand the internal linkages and differences between regions. This study has important implications on the coordinated formulation of inter-regional emission reduction policies.

## 3. Model Construction and Variable Selection

### 3.1. The Model Construction of ANTCE

The association network for transportation carbon emissions (*ANTCE*) is a collection of interrelationships between regional transportation carbon emissions. *ANTCE* is a complex network composed of nodes and lines. The “nodes” in the network denote the provinces of China, and the “lines” denote the spatial association of the transportation carbon emissions between provinces. Together, these form a spatial association network for inter-provincial transportation carbon emissions. In general, the network is constructed by using a vector auto regression (VAR) model and gravity model [23].

A gravity model can describe the strength of the connection between two regions, by combining geographical distance, economic development, population, and other factors. In particular, it can describe the evolutionary advantages in spatial associations using cross-section data. Using Borgatti’s research [24], we applied the gravity model to analyze the associations between provincial transportation carbon emissions, as shown in Equations (1) and (2):

(1) $$X_{ij}=\alpha_{ij}\frac{\sqrt{P_{i}C_{i}G_{i}}\sqrt{P_{j}C_{j}G_{j}}}{D_{ij}^{2}}$$

(2) $$\alpha_{ij}=\frac{c_{i}}{c_{i}+c_{j}},\;D_{ij}^{2}=(\frac{d_{ij}}{g_{i}-g_{j}}{)}^{2}$$

where *X_ij_* indicates the relationship between the transportation carbon emissions of provinces *i* and *j*; $a_{ij}$ presents the contribution rate of the provincial transportation carbon emissions flow (the proportion of the total carbon emissions of the two provinces being contributed by the transportation carbon emissions of province *i*). Transportation carbon emissions are asymmetry, so we corrected the gravity model by $a_{ij}$ and highlight the directionality of network connections between provinces *i* and *j*; $P_{i}$ is the proportion of the urban population in province *i*; $c_{i}$ indicates the carbon emissions in province *i*; $G_{i}$ is the GDP of province *i*; $d_{ij}$ indicates the linear distance between the capitals of provinces *i* and *j*; ($g_{i}-g_{j}$) indicates the difference in GDP per capita between provinces *i* and *j*; and D represents the “distance” between provinces *i* and *j*, defined by the ratio of $d_{ij}$ to ($g_{i}-g_{j}$).

Based on Equation (1), $X={\left(x_{ij}\right)}_{30\times 30}$ represents the gravity matrix between provinces. We used the average of the values in each row in the matrix as the “row threshold,” and then compared each value in the row to the row’s threshold (or average value). If an individual value in the row exceeded the row’s threshold (i.e., it was above the average), it was recorded as 1. This indicates that the provincial carbon emissions of that value in the row have a relationship with the corresponding column. Otherwise, the assigned value was 0. This approach generated the *ANTCE* matrix [25].

### 3.2. The Selection of Characteristic Indicators

#### 3.2.1. The Integral Network Analysis of the *ANTCE*

The integral network of the *ANTCE* represents the relationship network of the transportation carbon emissions between provinces in China. The characteristic indicators of the integral network of the *ANTCE* include the network density, network association degree, network grade, and network efficiency [26].

(1) The network density (*ND*) indicates the tightness of transportation carbon emissions between provinces. The calculation method is shown in Equation (3):

(3) $$ND=\frac{D}{N\left(N-1\right)}$$

where *ND* is the network density; *D* is the number of actual network relationships; *N* is the number of network nodes; and *N* (*N* − 1) is the maximum possible network relationship (i.e., the maximum number of directed segments between nodes). The closer the *ANTCE* network density is to 1, the closer the relationship between provincial transportation carbon emissions will be.

(2) Network association degree (*NAD*) is an indicator that reflects the robustness and vulnerability of the network structure. Equation (4) shows the calculation method. If all the areas of the *ANTCE* are directly connected, the inter-provincial transportation carbon emission network is more robust and the *NAD* will be higher. If multiple provinces are connected only through one region and if the carbon emissions of the region change or fluctuate, there will be a significant impact on *ANTCE*. This indicates that the network is fragile and *NAD* will be lower:

(4) $$NAD=1-\frac{V}{n(n-1)/2}$$

where *NAD* is the network association degree; *V* is the logarithm of the unreachable nodes in the network; and *n* is the number of provinces directly associated with other provinces.

(3) The network grade (*NG*) reflects the dominance of network members in the network [27]. Equation (5) shows the calculation method. The higher the network grade is, the more distinct the class is between provinces, and the more difficult it is to integrate high-carbon regions with low-carbon regions. This indicates an uneven *ANTCE* distribution:

(5) $$NG=1-\frac{R}{\mathrm{Max}(R)}$$

where *NG* is the network grade; R is the logarithm of the symmetric reachable node in the network; and max(R) is the logarithm of the largest possible reachable nodes.

(4) The network efficiency (*NE*) indicates the degree of redundant associations in the network. Equation (6) shows the calculation method. In the *ANTCE*, the lower the network efficiency, the more robust the *ANTCE* will be. This indicates redundant connections and spillover channels between provinces [26]:

(6) $$NE=1-\frac{K}{\mathrm{Max}(K)}$$

where *NE* is the network efficiency; *K* is the number of excess lines in the network; and *Max*(*K*) is the maximum number of possible excess lines in the entire network.

#### 3.2.2. The Centrality Analysis of the *ANTCE*

Centrality is an indicator used to measure the status and function of network nodes in *ANTCE* [28]. The region at the center of the *ANTCE* network has strong independence in transportation carbon emissions (i.e., this node has strong influence and control in the network), while the regions in marginal locations are mainly affected and controlled by the central region.

The centrality of the *ANTCE* network includes point centrality (*PC*) and betweenness centrality (*BC*). Point centrality (*PC*) directly reflects the status and power of the nodes in the *ANTCE* network [29]. Equation (7) presents the calculation method of the point centrality. In the directed graph, the point centrality of each node is further divided into in-centrality (the number of income associations of carbon emissions) and out-centrality (the number of spillover associations of carbon emissions):

(7) $$PC=\frac{n}{N-1}$$

The betweenness centrality (*BC*) refers to the node’s ability to control the relationship between other nodes [30]. Equation (8) is used to represent the *BC*. If a province is located in a connection position in the *ANTCE* network, the province has the ability to control the relationship of transportation-driven carbon emissions between other provinces:

(8) $$b_{jk}(i)=g_{jk}(i)/g_{ik}$$

(9) $$BC=\frac{2\sum_{j}^{N}\sum_{k}^{N}b_{jk}(i)}{3N^{2}-3N+2}$$

where $g_{jk}$ represents the number of shortcuts between nodes *k* and *j* in Equation (9); $b_{jk}(i)$ indicates the control ability of node *i* to node *k* and *j*, that is, the probability that *i* is in the shortcut between node *k* and *j*. The number of shortcuts passing through node *i* lying between node *k* and *j* is $g_{jk}(i)$, then $b_{jk}(i)=g_{jk}(i)/g_{jk}$, where *k* ≠ *j* ≠ *i*, and *j* < *k*.

#### 3.2.3. Spatial Aggregation Analysis

An aggregation subgroup is the nodes subset with relatively strong, close, and positive relationships in the *ANTCE* network, which is an expression for network aggregation attributes. The aggregation subgroup can be measured from the reciprocity, the proximity or reachability between the subgroups, the frequency of the relationship, the density of the relationship, etc. [31]. The aggregation reflects the aggregation characteristics of the spatial correlation network of provincial carbon emissions. Through the analysis of network aggregation, the overall network can be examined from a new perspective of internal block structure. Spatial aggregation can be used to segment the network, then the role of each block in the network can be further studied.

The block model is a method that partitions elements based on structural information about the network. Simplifying a complex network into a block model or an image matrix allows us to visually analyze the role and function of each part in the network [32]. The blocks in the network are generally divided into net spillover, two-way spillover, net income, and broker blocks [33]. (1) In the net spillover block, the provinces have more relationships with those provinces in other blocks than that of their own provinces, and do not receive many external relationships. (2) In the two-way spillover block, the provinces have no connection with other blocks. (3) In the net income block, the provinces obtain both relationships from external provinces and relationships from their own provinces. (4) In the broker block, the provinces both send and accept external relations, and there are fewer links between the internal provinces. The type of the block reflects the internal structure of each block, and the results of condensation in different regions. In this study, we grouped the provinces with similar position and functioning in the *ANTCE* into the same block. Then, we calculated the ratio of the expected and actual relationship between the provinces within this block, and the actual external relationship between this block and another block. This led to the determination of the role of the block. Wasserman and Faust [34] developed an indicator system to evaluate the internal relationships of locations (see Table 1). Based on this, we analyzed the types of each block by the division rules of the block model shown in Table 1. The $g_{k}$ indicates the number of provinces in block *k*, and *g* represents the number of all provinces in the *ANTCE* network.

### 3.3. QAP Regression Analysis of *ANTCE* Network

Collinearity may occur when studying the influencing factors of *ANTCE* due to the adjacent relationship between the provinces [35]. QAP, a method that compares the similarities of values in the two square matrices, can avoid this effect. The QAP method generates an association coefficient between two matrices by comparing the values of the matrices. This method also provides further non-parametric testing of the coefficient [36]. The purpose of QAP regression is to study the regression relationship between the influence matrix and *ANTCE*, and to evaluate the significance of the *R*^2^, as shown in Equation (10):

(10) $$R=f\left(X_{1},X_{2},X_{3},\ldots ,X_{n}\right)$$

where *R* refers to the *ANTCE* matrix, and *X_i_* (*I* = 1, 2, …, *n*) is the influence matrix. This matrix indicates the effect of carbon emissions and regional differences, and is an explanatory variable in the context of ordinary regression.

Currently, most studies have used spatial econometric methods to explore the factors influencing China’s inter-provincial carbon emissions. These methods have indicated that carbon emissions have been affected by geographical factors [37]. In addition, the block model results show that there are both direct and indirect spillovers in the eastern and western regions. Therefore, the transportation-related carbon emissions spillover may be related to the development mode of each region. The association of the *ANTCE* can be studied by measuring economic development differences in different regions. Therefore, we selected energy consumption, consumption levels, and urbanization indicators to indirectly describe the differences in the comprehensive development of each region.

Based on the spatial econometric analysis results of the factors affecting China’s transportation-driven carbon emissions [38], Equation (11) shows that we assumed that the influencing factors included: The spatial adjacency relationship (*SAM*), per capita GDP (*PAG*), fixed transportation investment (*TFI*), passenger turnover (*PAT*), freight turnover (*FRT*), urbanization rate (*UBR*), and energy utilization rate (*EUR*):

(11) T=f(SAM, PAG, TFI, PAT, FRT, UBR, EUR)

where *T* represents the spatial association matrix of transportation carbon emissions; *SAM*, *PAG*, *TFI*, *PAT*, *FRT*, *UBR*, and *EUR* are all relational matrices. *SAM* is the spatial adjacency matrix (if the two regions are adjacent, the value is 1, otherwise 0). We calculated the average of the indicators for each province during the study period (2007–2016), and then established the difference matrix by using the absolute difference of the corresponding indicators of each province in the *ANTCE*. The regression variables are represented by the relationship matrix representing the two regions. As such, it was impossible to use the general statistical test method to test whether there is a relationship between variables. Therefore, we selected the QAP correlation analysis and regression analysis method, which is a commonly used non-parametric method in social networks [39].

## 4. Empirical Analysis and Results Discussion

### 4.1. The Provincial Transportation Carbon Emission

**Figure 1:**
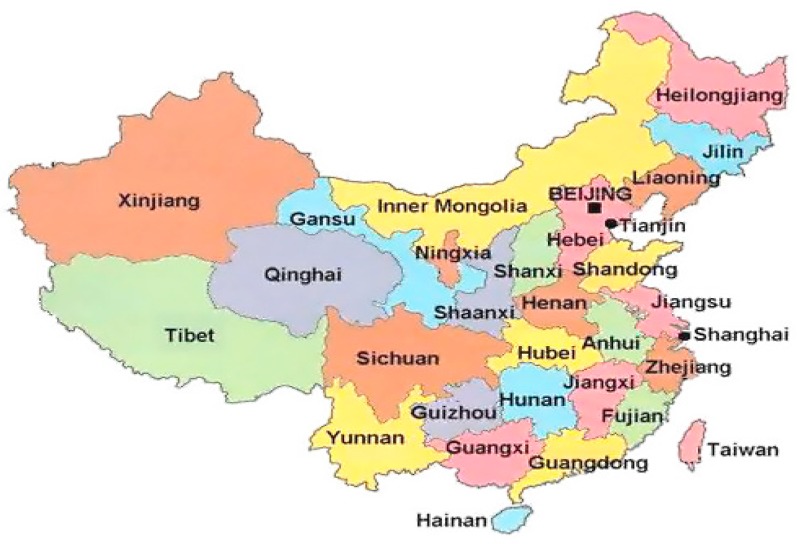
Research area.

This study selected 30 provinces in China as network nodes to model the *ANTCE* (see Figure 1). The data during 2007 to 2016 were used to calculate the provincial transportation carbon emissions. Then, we established the spatial relationship matrix of provincial transportation carbon emissions based on the modified gravity model. This study used the data of terminal energy consumption to calculate the carbon emissions, considering the accuracy of carbon emission estimates [40], as shown in Equation (12).

(12) $$C=\sum_{i-1}^{m}E_{i}\times ALV_{i}\times V_{i}\times r_{i}\times \frac{44}{12}$$

where *i* represents a different energy source; *ALV_i_* is the low energy calorific value of the energy source *i*; *V_i_* is the carbon content of the energy source, *i*; *r_i_* is carbon oxidation rate of the energy source *i*; *E_i_* is the energy consumption of the energy source, *i*; and *C* is the carbon dioxide emissions. The average low calorific value, the carbon of unit calorific value, and the carbon oxidation rate of the energy are shown in Table 2.

Provincial energy consumption data from the transportation industry were collected from the China Energy Statistical Yearbook (2007–2016) and the Provincial Statistical Yearbooks (2007–2016). Table 3 shows the provincial transportation carbon emissions in 2007 to 2016.

**Figure 2:**
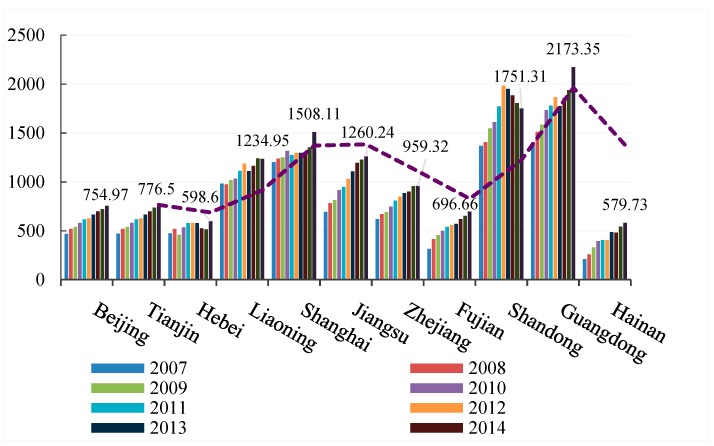
Transportation carbon emissions in the eastern region (2007–2016).

**Figure 3:**
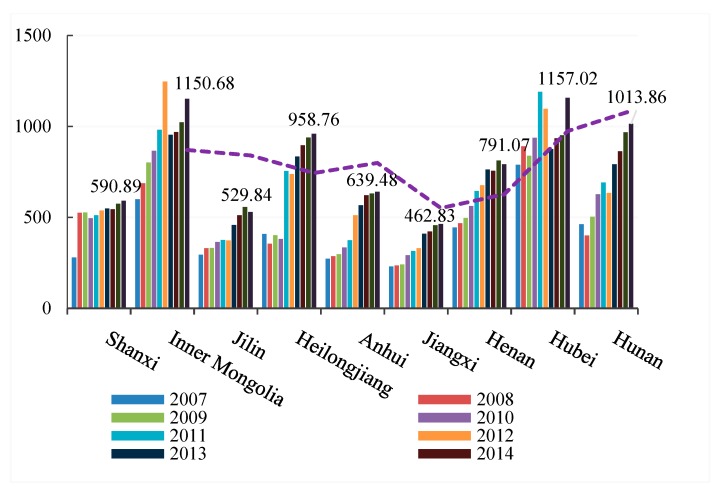
Transportation carbon emissions in the central region (2007–2016).

**Figure 4:**
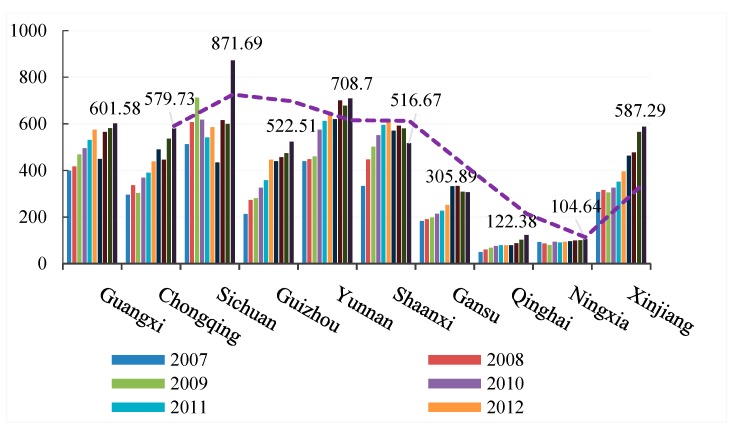
Transportation carbon emissions in the western region (2007–2016).

Table 3 shows that the provincial transportation carbon emissions rose from 2007 to 2016. To clearly express the peak changes of provincial carbon emissions in different years, we divided all provinces into eastern, central, and western regions for visual representation. Figure 2, Figure 3 and Figure 4 show that the transportation carbon emissions have increased in most provinces in 2016. In particular, Guangdong Province grew from 14.3 million tons in 2007 to 2173.35 million tons in 2016, at almost a rate of 1 million tons per year. From a provincial perspective, there were significant variations in the carbon emissions of the eastern, central, and western regions. The average levels of transportation carbon emissions in Shanghai, Shandong, and Guangdong were 10.6 times that of Gansu, Qinghai, and Ningxia.

From a regional perspective, the transportation carbon emissions were polarized, with significant differences in the carbon emissions in the eastern, central, and western regions. However, in 2015 to 2016, with the deep strategic development of the western region and the rise of the central region, the transportation-related carbon emissions in the central and western provinces increased rapidly.

### 4.2. Characteristics Analysis of the ANTCE

#### 4.2.1. Spatial Spillover and Autocorrelation Test

Traffic volume is one of the main factors in the change of transportation carbon emissions. Therefore, in this study, it was assumed that the functional relationship between transportation volume and transportation carbon emissions in each province is as follows:

(13) $$T{\mathrm{ce}}_{i}=\alpha +\beta Tve_{i}+\mu_{it}$$

where *T*ce_i_ is the transportation carbon emissions of each province during 2007 to 2016, *Tve_i_* is the traffic volume of the provinces during 2007 to 2016, and *μ_it_* is the error term. *α* and *β* are the regression coefficients of the econometric model.

If the spatial dependence of a variable in different regions is not tested and the econometric model is thereafter directly established, this may lead to the "pseudo-regression" phenomenon [43]. Therefore, the autocorrelation test must be first tested. We added a spatial weight matrix to the model. Under the first-order adjacency, we used Eviews to perform Ordinary Least Square (OLS) estimation on the simple linear regression model (13), and checked whether there was a spatial spillover effect. The results are shown in Table 4.

At the 1% level of significance, they are all significant. Therefore, it can be deduced that there is spatial association and spatial spillover effects. On this basis, we furtherer studied whether there is spatial autocorrelation. The Moran index is a statistical analysis technique that analyzes the spatial autocorrelation between regions [44]. This study uses spatial autocorrelation analysis to consider the relationship between two-dimensional space and carbon emissions before constructing a spatial association network. We used the Moran index obtained by Geoda to judge the spatial aggregation. The data is from the transportation carbon emissions of the provinces from 2007 to 2016.Overall, the province’s carbon emissions autocorrelation showed an overall upward trend from 0.0416 in 2007 to 0.1183 in 2016 with an increase rate of 184%, Table 5 shows the calculation results. This indicates that the regional association was gradually increasing as a whole, and regional carbon emissions were enhanced by the influence of surrounding areas.

#### 4.2.2. Spatial-Temporal Evolution Analysis of the *ANTCE*

**Figure 5:**
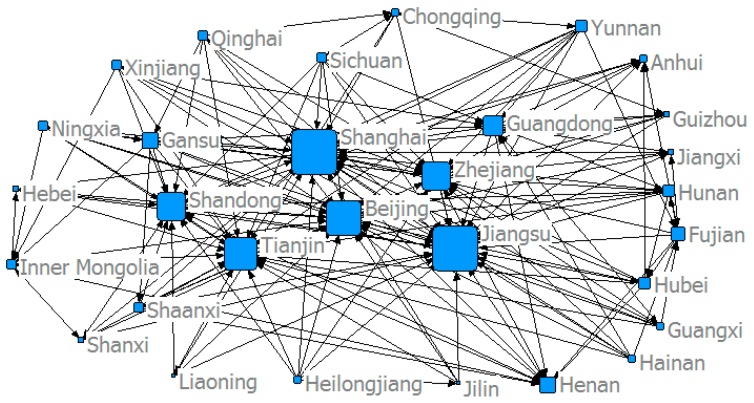
The structure of the *ANTCE* in 2007.

**Figure 6:**
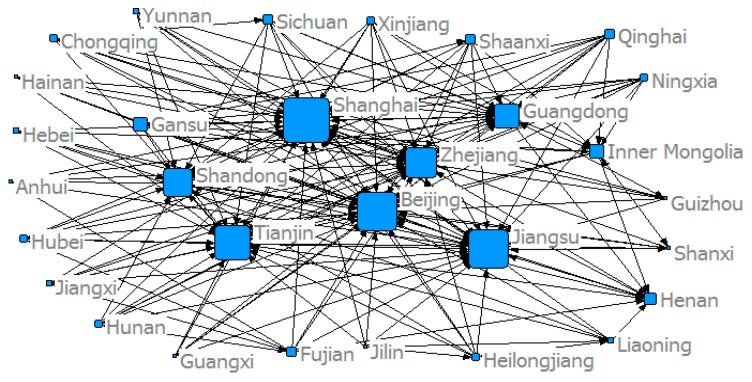
The structure of the *ANTCE* in 2010.

**Figure 7:**
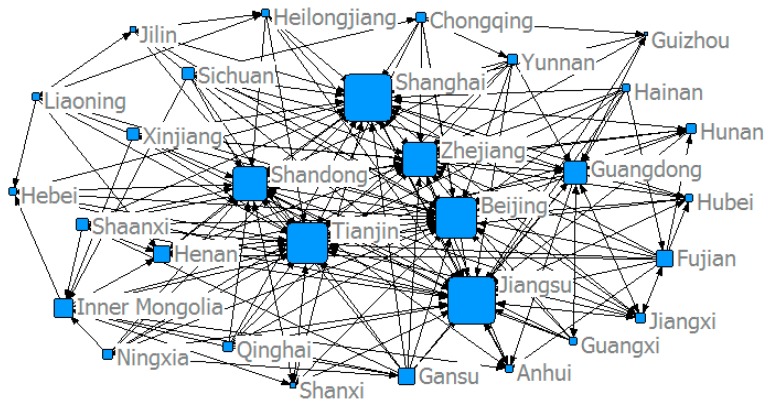
The structure of the *ANTCE* in 2013.

**Figure 8:**
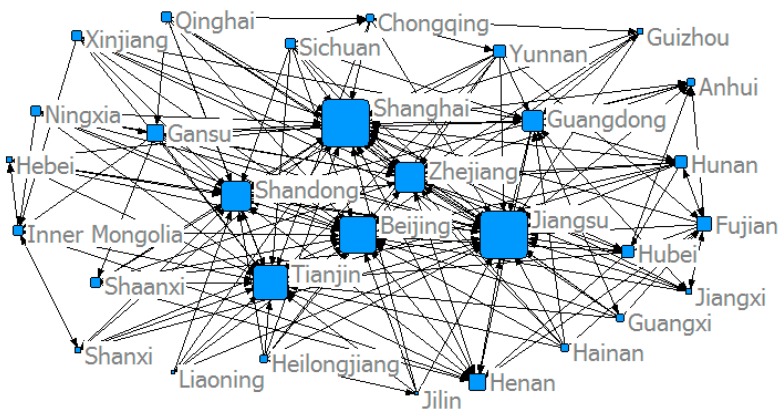
The structure of the *ANTCE* in 2016.

This study used the modified gravity model to determine China’s provincial *ANTCE* network, and established a spatial association matrix. Figure 5, Figure 6, Figure 7 and Figure 8 show the spatial network structures of the *ANTCE* in four specific years (2007, 2010, 2013, and 2016). The provincial transportation carbon emissions present a complex spatial network structure and spatial spillover relationship in the space network. The association of the marginal provinces was low. As such, the integral network did not show a completely closed spatial network. Therefore, there remained room for the development of the spatial association network. The network structure showed a clear decreasing trend in the network association in the “eastern developed provinces—central provinces—remote western provinces” after 2007. Beijing, Shanghai, Tianjin, Shandong, Zhejiang, and Jiangsu demonstrated strong outward radiation and control capabilities, and had a strong distribution and transfer effect on carbon emission flows. In contrast, the central and western provinces are scattered and located at the margin of the *ANTCE* network. It shows that there is an agglomeration effect on the network. Beijing, Tianjin, and Shanghai are closely related to other provinces in transportation carbon emissions. However, since 2013, the area of the nodes at the edge of the network has increased, indicating that the carbon emissions of transportation have increased. These provinces have begun to be closely related to other provinces rather than Beijing and Shanghai. The formation of such network distribution characteristics is closely related with the development strategies, such as “Western Development”, “Revitalization of Northeast China,” and “Beijing-Tianjin-Hebei Integration” and their corresponding geographical features [45].

(1) Network Density and Network Association in the *ANTCE*

**Figure 9:**
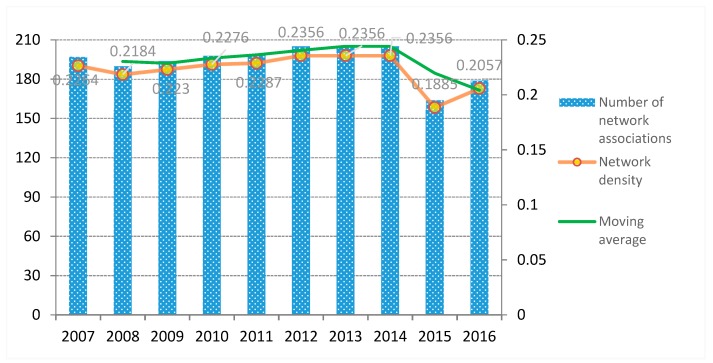
The density and the number of network association in the *ANTCE* network.

The *ND* value reflects the tightness of transportation carbon emissions between provinces [46]. The closer the *ND* value of the *ANTCE* is to 1, the closer the relationship between provinces was found to be. The robustness of *ND* in the *ANTCE* is concerned with the number of network associations (i.e., the number of connections between provinces). The more network associations there are, the better the robustness will be. The number of network associations first rose and then decreased during the study period (see Figure 9).

The number of network associations stabilized after 2012, reaching 176 in 2016. The average actual network association was 179. The *ND* also showed an “*N*” trend, indicating “first growing, then declining and then growing” rising from 0.179 in 2007 to 0.225 in 2016. This network belonged to the low-density network compared to the upper limit value of 1; the inter-provincial transportation carbon flow relationship was not close, whereas the network structure was relatively close. Although the *ND* value increased during the study period, the maximum number of associations was 205 from 2012 to 2014. The maximum possible spatial correlation remained very different, indicating significant opportunities to strengthen the spatial correlation of transportation-driven carbon emissions.

From a national policy perspective, China announced a number of carbon trading pilot areas in 2014. These areas successively issued a series of regulations in combination with their own economic development of the province, effectively reducing transportation-related carbon emissions. Thereafter, the traffic amount in 2015 slowed down since 2014. All the measures above led to a significant drop in the number of carbon emission network associations and network density in 2015.

(2) The *NG* and *NE* in the *ANTCE*

The *NAD, NE,* and *NG* values were used to analyze the spatial network association. The measurement results of *NAD* show that the *NAD* of the *ANTCE* was 1 during the sample period. This indicates direct or indirect links in the transportation-driven carbon emissions between provinces, and that the carbon emissions between provinces are closely related. There were two declines in the *NE* and *NG* values in 2012 and 2015. The network grade showed an overall W-shaped trend (see Figure 9).

**Figure 10:**
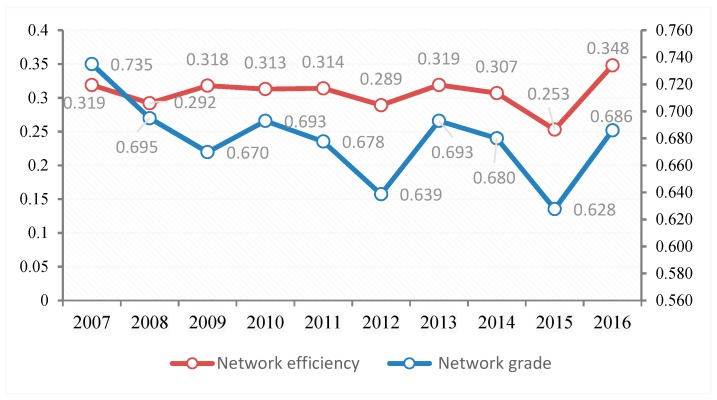
The efficiency and grade of the *ANTCE* network.

Figure 10 shows that the *NE* and *NG* values of provincial transportation carbon emissions experienced a downward trend. The *NG* in 2013 and 2015 dropped significantly, indicating a growth in the mutual influence of the *ANTCE*. The spatial association between the provinces in the *ANTCE* gradually increased, indicating that the provinces were breaking the previous strict hierarchical pattern. The inter-provincial carbon emissions flowed along a gradient and spread outward. Although the *NG* increased in 2016, it exhibited a declining trend. This indicates that the *ANTCE* structure was relatively stable.

The *NE* values were consistent with the trend of the network gradation, showing a downward trend. That is, the connection between *ANTCE* networks was closer than before. There was a certain proportion of redundant connections in the network, indicating a superposition of transportation carbon flow between provinces. This outcome also indicates that the spatial association of inter-provincial transportation carbon emission has become increasingly close, weakening the network structure with a significant gradient pattern.

#### 4.2.3. The Centrality Analysis of the *ANTCE*

The centrality of the *ANTCE* did not fluctuate over the last 10 years of the study period. As such, we only selected the point centrality (*PC*) and betweenness centrality (*BC*) of each province to conduct a central analysis of the *ANTCE* for 2016. Table 6 shows the calculation results.

Most provinces with high *PC* values are located in the eastern developed areas. The benefit-related relationship of these provinces is greater than the spillover relationship. This indicates that these areas have large carbon emissions and large transportation-related energy consumption. The flow of resources from other provinces to these areas is reflected in the spillover of carbon emissions. The provinces ranked lower in the sample period include Yunnan, Xinjiang, Hainan, and Ningxia. This indicates that these provinces have lower association levels with other provinces due to their relatively remote geographical location. This results in a weak association with other provinces.

Beijing, Zhejiang, and Guangdong had the top three *BC* values in 2016. This indicated that the linkage of transportation-related carbon emissions was mainly achieved through several developed regions. Most of the provinces with above average annual *BC* values were in the eastern developed regions during 2007 to 2016. The *BC* values of the lower ranked provinces were essentially 0. Most of these provinces were in the central and western region, where the transportation industry is underdeveloped. These areas have difficultly influencing the transportation carbon emissions of other provinces. All the *BC* values were different, indicating that there are unbalanced features.

#### 4.2.4. Spatial Aggregation Analysis of the *ANTCE*

**Figure 11:**
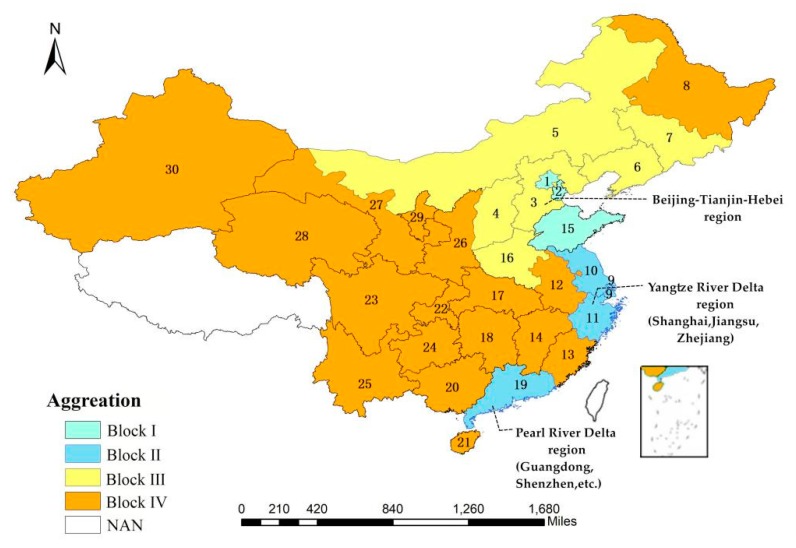
Block distribution of China’s provincial transportation carbon emissions.

From the results of spatial aggregation, Tianjin-Beijing-Hebei, the Yangtze River Delta, and the Pearl River Delta are closely related. The developed cities in the blocks I, II are equally attractive to prefecture-level cities in each province, which form a two-way overflow between most provinces. In addition, some provinces are in the transportation hub or geographical necessity. For example, Henan Province is located in the brokerage section and acts as a “bridge” in the whole network, which not only receives the overflow of the provinces in other blocks but also has connection with other provinces. Therefore, they are likely to have strong spatial connections of transportation energy with developed provinces, and the external spillover effect is significant (see Table 7 and Figure 11).

Table 8 shows the locations and properties of the four blocks in the spatial network associated with transportation-related carbon emissions. In 2016, there were 179 association relationships in the integral network of the *ANTCE*, including 21 and 158 association relationships within and between the blocks, respectively. This indicates that the provincial transportation carbon emissions between the blocks have a significant spatial association and spillover relationship. Table 8 shows that both block I and block II are consistent with “net income” and “two-way spillover” blocks. Blocks III and IV belong to the “broker” block. From the number of the sending and receiving, block I had a total of 58 receiving relationships and 18 transmission relationships. Block I had a spillover effect on both inside and outside the block, which belonged to the “two-way spillover block”. Block II had a total of 82 reception relationships and 20 transmission relationships. The number of relationships received by block II was significantly larger than the number of transmitted relationships, which was a “net income block”. Therefore, block I belongs to the two-way spillover, and block II belongs to the net benefit block. The total spillover of block IV and the relationship between the blocks exceeded those of block III. Therefore, block III belongs to the “broker” and block IV belongs to the “net spillover.”

First, the Bohai Rim, the Yangtze River Delta, and the Pearl River Delta region, represented by blocks I and II, are the regions with the highest level of economic development in China. The transportation modes are complete, possess large traffic volumes, and large levels of transportation energy consumption. These provinces require energy input from other resource-rich provinces. As a result, transportation carbon emissions from other provinces spill over into block I and II regions. Second, block III and Block IV represent areas with rich petroleum, coal, and natural gas reserves. Economic development is relatively slow in these areas. The road network conditions are poor, with insufficient road construction. As such, transportation-related carbon emissions are low in these areas.

In particular, the division of the block is related to the economic structure and transportation structure of the region rather than geographic location. For instance, Heilongjiang, Jilin, and Liaoning are located in Northeast China, but they belong to different blocks. Heilongjiang is classified to block IV, while Jilin and Liaoning to block III. Liaoning belongs to the Bohai Sea Economic Zone, which is the trade link between Northeast China and North China. It has a high level of economic development and a high concentration of traffic lines. In Jilin, the heavy industry accounts for a relatively high proportion of its gross output value, and road transportation accounts for a high proportion of transportation. The export of automobiles and agricultural products accelerates Jilin’s transportation carbon emissions. Therefore, Jilin and Liaoning belong to block III. However, the population of Heilongjiang has been at a low level, and due to its geographical and environmental constraints, transportation resources are also at a low level. Hence, Heilongjiang is included in block IV.

From the results of spatial aggregation, Tianjin-Beijing-Hebei, the Yangtze River Delta, and the Pearl River Delta are closely related. The developed cities in the blocks I, II are equally attractive to prefecture-level cities in each province, which form a two-way overflow between most provinces. On the other hand, due to the fact that some provinces are in the transportation hub or geographical necessity, for example, Henan Province is located in the brokerage section and acts as a “bridge” in the whole network, which not only receives the overflow of the provinces in other blocks but also has connection with other provinces. Therefore, they are likely to have strong spatial connections with developed provinces. The relationship of the rest of the provinces are becoming more close to the developed provinces with the output of transportation energy, and the external spillover effect is significant.

To investigate the relationship between the blocks, the density matrix was calculated based on Table 3. The results are shown in Table 8. For example, the integral ND was 0.2057 in 2016. When the ND of a certain block exceeded 0.2057, it indicated that the network density of the block was greater than the density level of the integral network. The block had a concentrated trend, and the value of the matrix was assigned 1 (the spatial network of provinces is more closely related in the block). Otherwise, the value was 0. Using this rule, we generated an image matrix [47] as shown in Table 9. When simplifying complex networks, the values in the initial matrix were rearranged to form a series of equivalent image matrices by a cluster analysis method. The image matrix visually reflects the spillover effect of transportation carbon emissions among the different blocks. This allows for a clearer examination of the transmission mechanisms associated with the transportation-driven carbon emissions. The matrix shows that block I and block II had a carbon emission association within their own block, and received spillover from blocks III and IV. There was also a spillover within block I and block II.

### 4.3. QAP Regression Analysis of Spatial Association Factors of the ANTCE

After analyzing the *ANTCE* and spatial aggregation characteristics of provincial transportation carbon emissions, it can be seen that China’s transportation carbon emissions are affected by geographical factors obviously, and there is obvious spatial aggregation. The eastern provinces with superior geographical position and developed economic development are more closely related, while the degree of network association between the central provinces and the remote western provinces is decreasing. The QAP regression method was used to further explore the factors affecting the spatial correlation of provincial carbon emissions, and analyze the factors influencing the *ANTCE*. The factors affecting carbon emissions mainly include transportation impact, economic development, and the scientific and technological level in the transportation industry [48]. Since the passenger and freight turnover (*PAT*, *FRT*) can directly affect the total amount of carbon emissions, it is determined to be a transportation factor; per capita GDP (*PAG*), transportation fixed investment (*TFI*), and urban rate (*UBR*) are indicators of economic development; the energy utilization rate (*EUR*) can reflect the technical development level of the provincial transportation industry; and the regional relationship is represented by a spatial adjacency matrix (*SAM*).

#### 4.3.1. QAP Association Analysis

The QAP association analysis of the spatial association matrix (T) and the influencing factors matrix was obtained by selecting 10,000 random permutations, as shown in Table 10. The rows of the matrix and the corresponding columns were simultaneously and randomly replaced. The correlation coefficient between the replaced matrix and a different matrix was calculated. The association coefficient represents the relationship between the relationship matrix of the explained variable (T) and the explanatory variable (*SAM*, *PAG*, *TFI*, *PAT*, *FRT*, *UBR*, and *EUR*). The greater the absolute value of the association coefficient, the greater the corresponding explanatory variable influences the network associated with the spatial carbon emissions. The maximum and minimum values were the values of the actual correlation coefficients from 10,000 random permutations. Values of *p* ≥ 0 and *p* ≤ 0 indicated the probability that the correlation coefficient observed in 10,000 random permutations would be greater and less than the actual correlation coefficient, respectively.

Table 10 shows that the association coefficients of *SAM*, *PAG*, *UBR*, and *EUR* with T were significant at a 1% level; the association coefficients of *TFI*, *PAT*, and *FRT* with T were significant at a 5% level. The association coefficient of *TFI* and *EUR* with T was negative. This indicates that energy utilization and fixed transportation investment are important factors influencing the spatial relationship of carbon emissions and spatial spillover effects.

The QAP association analysis was further performed on these seven variables. The geographical adjacency relationship was significantly correlated with other variables in Table 11; the per capita GDP and urbanization rate were significantly correlated at a 10% level. Therefore, there may be a multi-collinear influence of these seven explanatory variables on the spatial association of carbon emissions. The QAP method can better address the problem of overlap between independent variables in the regression.

#### 4.3.2. QAP Regression Analysis

To determine the effect of the influence matrix of these seven factors on the *ANTCE*, a QAP regression was conducted using UCINET software. Table 12 shows the results.

In Table 12, the regression coefficient of TFI is significant at 5%, indicating that the difference in transportation investment between provinces has an important impact on the spatial association and spillover of carbon emissions. Furthermore, the spatial association and spillover of transportation carbon emissions between provinces is greater. The regression coefficient of EUR is significant at the level of 5%, indicating that the difference in energy consumption between provinces has an important impact on the spatial association and spillover of carbon emissions. The regression coefficient value is negative, indicating that the higher the similarity of energy consumption, the greater the spatial association and spillover effect of transportation carbon emissions between provinces; the regression coefficients of PAT and FRT are significant at 5%, indicating that the difference in transport levels also affects the spatial correlation of carbon emissions to a certain extent, and the regression association is positive. The bigger the difference in the industrial structure and consumption level between regions, the greater the spatial association and spillover of carbon emissions between provinces. The regression coefficient of the difference matrix, PAG and UBR, is significant at 1%, which indicated that the economic situation of the province has an important impact on the spatial network relationship of transportation carbon emissions, and the more developed the province, the greater the impact.

## 5. Conclusions and Policy Implications

### 5.1. Conclusions

This study applied the *SNA* method to explore the spatial association and factors influencing the *ANTCE*, leading to the following conclusions. From the perspective of integral network structure characteristics, network associations and network density increased during the study period, and there were significant spatial association and spatial spillover in the *ANTCE*. The carbon emission levels from transportation between provinces tended to be equal, and an increasing number of provinces were in a controlling position in the carbon emission network. The increase in the connection between provincial transportation carbon emissions made the connection closer. The space-related network was more complicated and stable. From the perspective of the characteristics of each node of the network, there was a different *BC* value for each province in the transportation carbon emission network, and the characteristics were unbalanced.

Spatial aggregation features (using the block model) showed that block I and II were mainly concentrated in the Beijing-Tianjin-Hebei region, the Yangtze River Delta, and Pearl River Delta regions. These provinces have developed economies, centralized transportation resources, complete transportation infrastructure, and numerous roads. This results in high transportation carbon emissions. Block III was mainly located in the North-Central region, playing the role of an intermediary media and bridge connecting the spatial network of carbon emissions. Block IV was mainly located in the remaining provinces, with abundant energy resources in the Central and Western remote areas.

The QAP regression analysis results show that spatial adjacency, energy utilization, industrial structure differences, and urbanization differences significantly impact on the spatial association of China’s transportation carbon emissions. Inter-regional energy efficiency and fixed investment in transportation inhibit transportation carbon emissions. The higher the two factors, the greater the spatial association and the spillover of carbon emissions between the provinces.

### 5.2. Policy Recommendations

Based on the conclusions above, this study proposes the following policy recommendations. First, the economically developed regions, such as Shanghai, Beijing, and Tianjin, can adjust the transportation energy structures, raise industry emission reduction standards, and conduct non-invasive market interventions. Remote provinces with rich resources should strengthen railway network construction and improve energy efficiency. The eastern region and some central and western regions are closely connected with other regions. As such, the transmission and the flow of transport carbon emissions in the network will be strengthened by vigorously building transport corridors. At the national level, the government can reduce carbon intensity by establishing a mechanism for sharing the responsibilities for transportation carbon emission reduction among provinces, and improving the green recycling and low-carbon traffic assessment system.

Second, from a spatial association effect perspective, China’s transportation carbon emissions show significant spatial clustering characteristics. The transportation-driven carbon emissions in this region are positively affected by neighboring provinces. Therefore, policies need to consider the traffic conditions, economic development, traffic carbon emission levels, location factors in the region, and the spatial interaction effects of traffic carbon emissions. The government should focus on reducing transportation-driven carbon emission in closely related areas. This could involve using the Yangtze River Delta provinces, such as Jiangsu, Zhejiang, and Shanghai, or Beijing-Tianjin-Hebei, as a demonstration area for emission reductions to radiate and drive emissions reduction work in surrounding areas.

Finally, local governments should optimize transportation system structures and improve transportation organization efficiency. This would involve focusing on adjusting transportation structure, reducing the proportion of highway transportation, and increase the proportion of low-emission transportation modes, such as railways, waterways, and civil aviation. 

This study has generated much research value in terms of the transportation of carbon emissions. However, it has some limitations. We adopted a top-down calculation method to decompose transportation carbon emissions but did not fully consider all emission errors caused by traffic flow between provinces. In addition, due to limited data, we constructed China’s transportation carbon emission network from a provincial perspective. City-level data, combined with geo-economics, would lead to a more accurate analysis of the spatial association between China’s regional carbon emissions. Therefore, future research should explore more data resources to improve the investigative and research methods in this research field.

## Figures and Tables

**Table 1:** Block model division in the *ANTCE* network.

Ratio within the Block	Ratio Received by Block	
	≈0	>0
$\geq \left(g_{k}-1\right)/\left(g-1\right)$	Two-way spillover	Net income block
$\leq \left(g_{k}-1\right)/\left(g-1\right)$	Net spillover block	Broker block

**Table 2:** Energy calculation coefficient.

Energy	Average Low Calorific Value ^1^ (KJ/kg)	Standard Coal Coefficient ^1^ (Kgce/kg)	Carbon of Unit Calorific Value ^1^ (tons of Carbon/TJ)	Carbon Oxidation Rate ^2^	Carbon Dioxide Emission Coefficient ^2^
Raw coal	20,908	0.7143	26.37	0.94	1.9003
Washed coal	26,344	0.9000	25.8	0.90	2.2400
Briquette	17,772	0.6000	25.8	0.90	1.5100
Coke oven gas	17,981	0.6143	12.1	0.99	0.7900
Other coking products	33,779	1.3000	15.7	0.98	1.9100
Coke	28,435	0.9714	29.5	0.93	2.8604
Fuel oil	41,816	1.4286	21.1	0.98	3.1705
Gasoline	43,070	1.4714	18.9	0.98	2.9251
Kerosene	43,070	1.4714	19.5	0.98	3.0179
Diesel	42,652	1.4571	20.2	0.98	3.0959
liquefied petroleum gas	50,179	1.7143	17.2	0.98	3.1013

Note: The data in footnote 1 comes from the General Rules for the Calculation of Comprehensive Energy Consumption (GB/T 2589-2008) [41]; the data in footnote 2 are derived from the Guidelines for the Preparation of Provincial Greenhouse Gas Inventories. (Climate Office [2011] No. 1041) [42].

**Table 3:** Provincial transportation carbon emissions during 2007 to 2016.

Province	Transportation Carbon Emissions (10,000 tons)									
	2007	2008	2009	2010	2011	2012	2013	2014	2015	2016
Beijing	466.93	520.48	539.72	581.83	616.92	625.98	664.11	698.40	720.99	754.97
Tianjin	470.96	520.48	539.72	581.83	615.39	626.84	664.11	698.40	735.63	776.50
Hebei	473.08	520.48	457.94	534.70	577.24	579.58	579.09	524.72	513.62	598.60
Shanxi	279.42	525.41	526.17	495.17	511.71	537.36	549.18	543.96	574.91	590.89
Inner Mongolia	599.58	686.35	800.36	865.99	980.37	1245.62	953.90	969.02	1022.37	1150.68
Liaoning	983.39	974.56	1018.61	1034.17	1113.69	1187.17	1109.86	1163.18	1239.61	1234.95
Jilin	294.95	330.21	332.15	364.88	376.12	372.50	458.17	512.21	556.62	529.84
Heilongjiang	407.82	354.53	402.25	380.98	754.10	737.86	833.86	896.27	938.88	958.76
Shanghai	1202.30	1238.02	1249.71	1316.36	1274.28	1296.27	1295.61	1290.61	1351.68	1508.11
Jiangsu	693.64	781.65	812.65	916.74	948.56	1030.14	1106.71	1194.25	1227.73	1260.24
Zhejiang	620.24	671.09	690.58	746.34	808.74	849.24	885.21	901.55	956.04	959.32
Anhui	271.90	286.46	296.94	334.35	373.55	511.88	566.71	621.36	630.85	639.48
Fujian	313.60	414.58	455.56	498.40	539.45	557.50	569.26	619.19	653.38	696.66
Jiangxi	230.18	234.76	240.74	291.03	315.64	330.66	409.35	421.68	456.52	462.83
Shandong	1367.89	1407.49	1546.64	1610.70	1771.02	1983.98	1952.53	1883.99	1804.89	1751.31
Henan	444.41	467.03	496.36	562.96	643.75	676.00	762.26	755.87	812.03	791.07
Hubei	788.99	890.82	837.72	937.09	1189.21	1096.25	874.70	934.17	950.96	1157.02
Hunan	462.10	400.30	502.84	626.76	690.64	632.91	790.90	862.74	966.85	1013.86
Guangdong	1403.42	1512.70	1585.32	1733.33	1780.59	1865.91	1774.75	1859.14	1937.97	2173.35
Guangxi	398.42	416.61	467.53	494.64	530.07	574.04	448.23	565.10	581.14	601.58
Hainan	209.40	258.52	330.03	394.79	402.96	405.38	487.22	481.03	542.92	579.73
Chongqing	295.65	336.22	302.79	368.62	390.02	437.56	489.24	445.32	535.79	579.73
Sichuan	512.30	607.06	711.54	617.57	540.95	585.23	434.09	615.46	598.68	871.69
Guizhou	213.28	272.87	280.23	325.29	358.35	445.20	439.27	456.03	473.45	522.51
Yunnan	439.52	447.94	459.30	574.01	611.90	651.63	618.98	700.40	677.44	708.70
Shaanxi	332.90	446.12	500.81	551.00	594.75	609.51	570.01	591.12	579.93	516.67
Gansu	182.79	190.77	198.40	213.91	226.70	251.06	332.17	332.88	308.65	305.89
Qinghai	49.48	60.68	68.01	75.18	78.47	79.08	78.85	86.73	110.09	101.38
Ningxia	92.47	86.40	79.54	93.81	90.69	93.38	95.37	99.36	100.34	104.64
Xinjiang	307.56	315.62	304.80	325.51	352.00	395.60	462.51	476.20	564.33	587.29

**Table 4:** Lagrange multiplier (LM) test result.

Year	LM (Lag)	LM (Error)	Robust LM (Lag)	Robust LM (Error)
2007	29.12 ***	38.63 ***	32.26 ***	40.18 ***
2008	31.81 ***	35.83 ***	33.95 ***	39.61 ***
2009	28.13 ***	37.58 ***	31.53 ***	40.07 ***
2010	29.45 ***	38.37 ***	33.74 ***	41.36 ***
2011	27.42 ***	34.29 ***	29.86 ***	36.71 ***
2012	28.16 ***	35.32 ***	32.97 ***	38.91 ***
2013	32.04 ***	41.35 ***	36.48 ***	45.39 ***
2014	28.75 ***	36.02 ***	32.13 ***	40.51 ***
2015	31.28 ***	40.68 ***	35.78 ***	45.18 ***
2016	30.69 ***	39.72 ***	34.69 ***	44.26 ***

Note: *** indicates significant at the 1% confidence level.

**Table 5:** The test of spatial autocorrelation of transportation carbon emissions.

Year	Moran’s I Value	Z-Value	*p*-Value
2007	0.0416	0.8276	0.182
2008	0.0275	0.6214	0.261
2009	0.0803	1.519	0.086
2010	0.1120	2.008	0.041
2011	0.1007	1.931	0.044
2012	0.1211	2.016	0.023
2013	0.1217	2.016	0.023
2014	0.1216	2.016	0.023
2015	0.1349	2.331	0.034
2016	0.1183	2.120	0.019

**Table 6:** The centrality analysis of the *ANTCE.*

Province	Point Centrality (*PC*)			Betweenness Centrality (*BC*)
	In-Centrality	Out-Centrality	Center Degree	Betweenness Degree
Shanghai	27	5	93.103	61.161
Beijing	24	7	82.759	145.522
Jiangsu	21	3	72.414	9.239
Zhejiang	19	4	65.517	113.067
Tianjin	18	5	62.069	20.044
Shandong	16	6	58.621	20.239
Guangdong	15	8	62.069	120.867
Henan	8	6	27.586	51.85
Anhui	6	3	20.69	6.167
Jiangxi	3	6	20.69	103.686
Liaoning	3	5	17.241	56.167
Inner Mongolia	3	4	20.69	0.417
Hebei	3	4	13.793	0.417
Hunan	2	6	24.138	1.985
Guizhou	2	5	20.69	1.292
Shanxi	2	5	17.241	1.417
Gansu	1	8	31.034	0.5
Fujian	1	8	27.586	7.7
Hubei	1	7	24.138	2.333
Sichuan	1	7	27.586	0
Heilongjiang	1	6	20.69	4.333
Guangxi	1	5	17.241	1.292
Jilin	1	5	17.241	0.333
Chongqing	0	9	31.034	0
Ningxia	0	8	27.586	0
Qinghai	0	8	27.586	0
Yunnan	0	7	24.138	0
Xinjiang	0	7	17.241	0
Shaanxi	0	7	24.138	0
Hainan	0	5	24.138	0

**Table 7:** Inter-provincial transportation carbon emission aggregation results.

Blocks No.	Provinces (with No.)
Block I	1 Beijing, 2 Tianjin, 15 Shandong
Block II	9 Shanghai, 10 Jiangsu, 11 Zhejiang, 19 Guangdong
Block III	3 Hebei, 4 Shanxi, 5 Inner Mongolia, 6 Liaoning, 7 Jilin, 16 Henan
Block IV	8 Heilongjiang, 12 Anhui, 13 Fujian, 14 Jiangxi, 17 Hubei, 18 Hunan, 20 Guangxi, 21 Hainan, 22 Chongqing, 23 Sichuan, 24 Guizhou, 25 Yunnan, 26 Shaanxi, 27 Gansu, 28 Qinghai, 29 Ningxia, 30 Xinjiang

**Table 8:** The spillover effects of spatial associations of the *ANTCE* block.

Block No.	Provinces Number	Receiving Relationship		Sending Relationship		Expected Internal Relationship Ratio	Actual Internal Relationship Ratio	Block Attribute
		Inside the Block	Outside the Block	Inside the Block	Outside the Block			
Block I	3	6	52	6	12	6.89	33.33	Two-way spillover
Block II	4	5	77	5	15	10.34	25	Net income block
Block III	6	3	17	3	26	17.24	10.34	Broker block
Block IV	17	7	12	7	105	55.17	6.25	Net spillover

**Table 9:** Density matrix and image matrix of the *ANTCE*.

Block No.	Density Matrix				Image Matrix			
	Block I	Block II	Block III	Block IV	Block I	Block II	Block III	Block IV
Block I	1.000	0.063	0.556	0.02	1	0	1	0
Block II	0.083	0.417	0.167	0.147	0	1	0	0
Block III	0.833	0.417	0.1	0.01	1	1	0	0
Block IV	0.706	0.971	0.029	0.026	1	1	0	0

**Table 10:** Analysis of the association matrix T and QAP of influencing factors.

Variable Name	Association Coefficient	Significant Level	Mean Coefficient of Correlation	Standard Deviation	Minimum Value	Maximum	*p* ≥ 0	*p* ≤ 0
SAM	0.212 ***	0000	0.044	−0.066	−0.187	0.188	0.000	1.000
PAG	0.153 ***	0000	0.059	−0.135	−0.236	0.196	0.000	1.000
TFI	−0.080 **	0.023	0.065	−0.106	−0.204	0.235	0.000	1.000
PAT	0.053 **	0.015	0.059	−0.133	−0.198	0.195	0.000	1.000
FRT	0.028 **	0.020	0.056	−0.137	−0.212	0.194	0.000	1.000
UBR	0.063 ***	0.003	0.048	0.023	−0.125	0.162	0.000	1.000
EUR	−0.050 ***	0.008	0.062	−0.156	−0.052	0.189	0.000	1.000

Note: *** indicates significant at the 1% level; ** indicates significant at the 5% level.

**Table 11:** The QAP association analysis of influencing factors.

Variable Name	SAM	PAG	TFI	PAT	FRT	UBR	EUR
SAM	1.000 ***	−0.153 ***	−0.094 **	−0.145 ***	0.053 **	−0.058 **	0.084 **
PAG	−0.153 ***	1.000 ***	0.037	0.125	0.135	0.234 *	−0.125
TFI	−0.094 **	0.037	1.000 ***	−0.087 **	0.066 **	0.093	−0.080
PAT	−0.145 ***	0.125	−0.087 **	1.000 ***	−0.107	−0.034	0.046
FRT	0.053 **	0.135	0.066 **	−0.107	1.000 ***	−0.035	0.006
UBR	−0.058 **	0.234 *	0.093	−0.034	−0.035	1.000 ***	−0.023
EUR	0.084 **	−0.125	−0.080	0.046	0.006	−0.023	1.000 ***

Note: * indicates significant at the 10% confidence level; ** indicates significant at the 5% confidence level; *** indicates significant at the 1% confidence level.

**Table 12:** The results of QAP regression analysis.

Variable Name	Non-Standardized Regression Coefficient	Standardized Regression Coefficient	Significant Probability Value	Probability 1	Probability 2
SAM	0.015	0.064 **	0.038	0.549	0.451
PAG	0.201	0.172 ***	0.000	0.000	1.000
TFI	−0.017	−0.027 *	0.066	0.700	0.301
PAT	0.052	0.011 *	0.082	0.632	0.368
FRT	0.064	0.017 *	0.081	0.534	0.466
UBR	0.025	0.000 *	0.067	0.639	0.362
EUR	−1.503	−0.045 ***	0.000	0.418	0.973

Note: ** indicates significant at a 5% confidence level; *** indicates significant at a 1% confidence level; Probability 1 denotes the probability that the regression coefficient is greater than or equal to the final regression coefficient during random replacement; Probability 2 denotes the probability that the regression coefficient is less than or equal to the final regression coefficient during random replacement.
